# Assessment of cardiac load-responsiveness in veno-arterial extracorporeal life support: A case series

**DOI:** 10.1177/02676591231181463

**Published:** 2023-06-06

**Authors:** Y Cornelisse, PW Weerwind, ME Bol, AP Simons

**Affiliations:** 1 Department of Extra-Corporeal Circulation and Cardiothoracic Surgery, 199236 Maastricht University Medical Centre, Maastricht, Netherlands; 2 Department of Intensive Care Medicine, 199236 Maastricht University Medical Centre, Maastricht, Netherlands; 3 School of Nutrition and Translational Research in Metabolism, 199236 Maastricht University Medical Centre, Maastricht, Netherlands; 4 Advanced Extracorporeal Therapies - perfusion services, training & education, Landgraaf, the Netherlands

**Keywords:** veno-arterial extracorporeal life support, centrifugal pump, blood volume, venous drainage, cardiac load-responsiveness

## Abstract

**Introduction:**

Well-timed explant of veno-arterial extracorporeal life support (V-A ECLS) depends on adequate assessment of cardiac recovery. Often, evaluation of cardiac recovery consists of reducing support flow while visualizing cardiac response using transoesophageal echocardiography (TEE). This method, however, is time consuming and based on subjective findings. The dynamic filling index (DFI) may aid in the quantitative assessment of cardiac load-responsiveness. The dynamic filling index is based on the relationship of support flow and pump speed, which varies with varying hemodynamic conditions. This case series intends to investigate whether the DFI may support TEE in facilitating the assessment of cardiac load-responsiveness.

**Methods:**

Measurements for DFI-determination were performed in seven patients while simultaneously assessing ventricular function by measuring the aortic velocity time integral (VTI) using TEE. Measurements consisted of multiple consecutive transient speed manipulations (∼100 r/min) during weaning trials, both at full support and during cardiac reloading at reduced support.

**Results:**

The VTI increased between full and reduced support in six weaning trials. In five of these trials DFI decreased or remained equal, and in one case DFI increased. Of the three trials in which VTI decreased between full and reduced support, DFI increased in two cases and decreased in one case. Changes in DFI, however, are mostly smaller than the detection threshold of 0.4 mL/rotation.

**Conclusion:**

Even though current level of accuracy of the parameter requires further investigation to increase reliability and possibly predictability, DFI seems likely to be a potential parameter in supporting TEE for the assessment of cardiac load-responsiveness.

## Introduction

Assessment of weanability, i.e. sufficient cardiac recovery, of veno-arterial extracorporeal life support (V-A ECLS) in patients suffering from acute cardiac failure is challenging, but remains crucial for positive outcome.^1–5^ A common procedure to assess weanability consists of reducing support flow while visualizing the cardiac response using transoesophageal echocardiogram (TEE).^4,6,7^ That support reduction addresses the Frank-Starling response by inducing an increased volume load on the myocardium. Based on TEE, cardiac function is determined by eyeballing cardiac wall motion and calculating the velocity time integral (VTI) over the aortic valve.^6,8^ This, however, is time-consuming and provides merely a situational snap-shot. Moreover, results are rather subjective, which consequently may lead to unnecessarily prolonged duration of support. As such, any additional method to assess cardiac recovery would be beneficial.

The cardiac assessment procedure as mentioned earlier affects blood volume distribution, which in patients with poor cardiac function is likely to result in venous congestion and increased stressed venous volume. Unfortunately, assessing venous volume, which could be an indicator of cardiac load-responsiveness and weanability, can be quite challenging as it is limited to the subjective interpretation of hemodynamic and pump-related parameters.^9–11^ However, Simons et al. introduced a technique to quantitatively assess venous volume that can be (potentially) drained by the centrifugal pump-based V-A ECLS circuit.^12,13^ The results of their studies showed the relationship of pump speed and resultant bypass flow, and was named the dynamic filling index (DFI). This index is modulated by a non-absolute volumetric number (drainable volume), and showed to be inversely related to cardiac function.^14^ As such, the DFI was defined as: DFI = Δbypass flow/Δpump speed, and can be determined by inducing small fluctuations in pump speed while measuring resultant changes in support.

This current case series intends to show whether the DFI may support TEE in facilitating the assessment of cardiac load-responsiveness during acute reloading.

## Methods

This explorative study was approved by the Joint Institutional Committee on Ethics of Human Investigation of the University Hospital Maastricht and Maastricht University and Central Committee on Research Involving Human Subjects (CCMO No. NL49011.000.14), and was in accordance with the World Medical Association Declaration of Helsinki. Patients were included after written informed consent was obtained from patients’ legal representatives or patient relatives.

**Figure 1.**
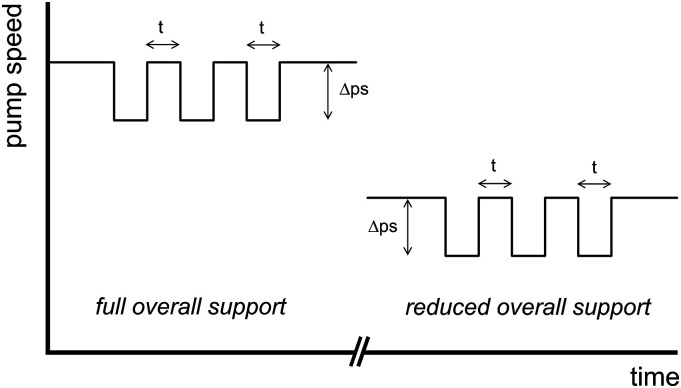
Example of multiple transient pump speed manipulations superimposed on the steady state pump speed at full overall support and at reduced overall support. t, duration of speed manipulation (ca. 10 s); ∆ps, amplitude of speed manipulation (ca. 100 r/min).

Seven patients assisted by V-A ECLS were enrolled. Measurements were performed during the weaning trials prior to and immediately after the coherent reduction in overall support flow (Figure 1). During a measurement at either full overall support or at reduced overall support, pump speed was manually and periodically manipulated to create sequences consisting of multiple consecutive transient speed reductions of approximately 100 r/min, each lasting approximately 10 s, superimposed on the steady state pump speed.

The following parameters were recorded upon inclusion: patient age, weight and length, and venous and arterial cannula type/size. Support flow and pump speed data were registered at 1.0 Hz using the pump console’s digital output. Ventricular function was assessed using VTI measurements based on TEE.

### Data processing and statistical analysis

Data processing was performed using MS Excel (Microsoft Corp., Redmond, WA, USA) and MATLAB r2020b (The MathWorks Inc., Natick, MA, USA). Statistical analysis was performed using the Statistical Package of Social Sciences (SPSS 27, IBM, Armonk, New York, USA).

For both V-A ECLS flow and pump speed the last three samples (3 s) preceding each speed manipulation (up and down) were taken and averaged. From the average per point the DFI values (mL/rotation) were calculated by dividing the measured change in overall support flow by the induced change in pump speed (DFI = Δflow/Δspeed). All DFI values measured in one period were averaged by taking the median value. The interquartile range of the DFI within one period was used to assess intra-sequence reproducibility.

The relationship between values for VTI and DFI was assessed by regression analysis. In addition, ∆DFI and ∆VTI were calculated by extracting the DFI and VTI values at full support from the corresponding DFI and VTI values at reduced support, respectively, and subsequently plotted.

## Results

Patients included (*n* = 7) underwent one or more weaning trials, resulting in a total of 14 weaning trials. In Table 1 the patient characteristics are shown. Five patients were cannulated femoro-femorally, whereas one patient was centrally cannulated. One patient had a combination of both, i.e. femoral-aortic cannulation. During weaning trials cardiac reloading was performed by reducing overall support flow by approximately 40–50%, from 4.0 ± 1.0 L/min to 2.2 ± 0.5 L/min on average.

**Table 1.** Patient demographics.

Parameter	Value
Male/female (n)	4/3
Age (years)	61 ± 8
Height (cm)	173 ± 11
Weight (kg)	84 ± 14

Values represent mean ± standard deviation.

**Figure 2.**
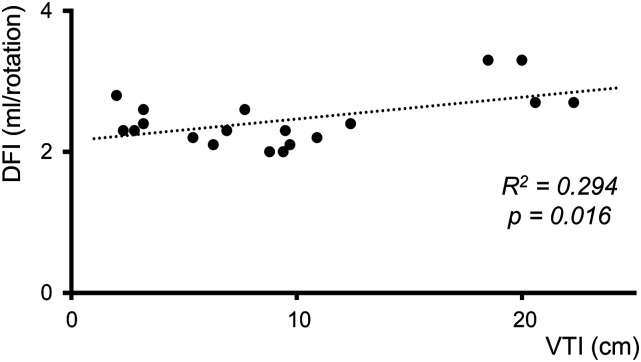
19 median dynamic filling indices plotted against the corresponding velocity time integrals that were derived from weaning trials. DFI, dynamic filling index; VTI, velocity time integral.

Table 2 displays the results of the weaning trials. The interquartile range within one measuring period varies from 0.0 mL/rotation to 1.1 mL/rotation, with an overall average of 0.4 mL/rotation. In five weaning trials the TEE recordings were of insufficient quality to determine VTI, resulting in 19 calculated VTI values. In Figure 2 the DFI values of all patients are plotted against the corresponding VTI values. A significant positive weak regression equation was found, *R*^2^ = 0.294, *F*(1, 17) = 7.096, *p* = 0.016.

**Table 2.** VTI and DFI results from the performed weaning trials.

		VTI		DFI (IQR)	
Weaning trial	Patient	Full overall support	Reduced overall support	Full overall support	Reduced overall support
1	A	–	–	2.3 (0.4)	2.9 (0.6)
2	C	–	–	2.1 (0.4)	2.0 (0.0)
3	C	–	–	2.4 (0.8)	2.0 (0.6)
4	C	9.4	–	2.0 (0.2)	2.0 (0.1)
5	D	–	–	2.5 (0.4)	2.8 (0.3)
6	D	3.2	2.0	2.4 (0.6)	2.8 (0.6)
7	D	6.9	9.7	2.3 (0.2)	2.1 (0.3)
8	E	5.4	12.4	2.2 (0.3)	2.4 (0.2)
9	E	10.9	9.5	2.2 (0.4)	2.3 (0.4)
10	F	3.2	2.8	2.6 (1.1)	2.3 (0.7)
11	F	7.7	8.8	2.6 (0.3)	2.0 (0.2)
12	G	20.6	22.3	2.7 (0.2)	2.7 (0.6)
13	G	18.5	20.0	3.3 (0.4)	3.3 (0.7)
14	H	2.3	6.3	2.3 (0.5)	2.1 (0.4)

VTI, velocity time integral; DFI, dynamic filling index; IQR, interquartile range.

**Figure 3.**
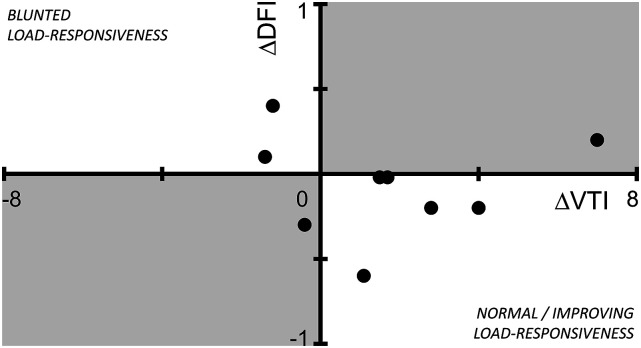
Scatter plot of ∆ velocity time integral (VTI) versus ∆ dynamic filling index (DFI). ∆DFI is inversely related to ∆VTI for weaning trials located in the lower right and upper left quadrant (normal/improving load-responsiveness and blunted load-responsiveness), whereas those located in the shaded areas (lower left and upper right quadrant) show ∆DFI to be directly related to ∆VTI.

For nine out of the 14 weaning trials (6–14) both a ∆DFI and ∆VTI could be calculated (Figure 3). ∆VTI increased in six of these nine trials. Of these six, five showed a decreased or similar ∆DFI, and one an increased ∆DFI. In three of those nine trials ∆VTI decreased, two showed an increased ∆DFI and one showed a decreased ∆DFI.

## Discussion

This report shows that current implementation of the DFI cannot support TEE in facilitating the assessment of cardiac load-responsiveness in patients assisted by V-A ECLS during acute reloading.

Several studies have investigated V-A ECLS weaning protocols and/or reported parameters for predicting successful weaning from V-A ECLS.^8,15–17^ These studies used either basic hemodynamically-derived or TEE-related parameters or a combination of both to gauge cardiac function. Retrieving those parameters, however, can be time-consuming, whereas read-outs merely provide a situational snapshot of the cardiac function *ad praesens*. Considering the ease-of-use of DFI measurements, which are based on continuously derived pump-related and cardiac function dependent hemodynamic parameters, DFI might aid in the assessment of cardiac function. Moreover, as it uses concrete values, DFI cancels out measurement subjectivity and may optimize weaning, especially when performed automated and in a regular fashion.

The DFI is a measure of on-pump drainable venous volume.^12^ When venous volume increases as a result of decreased cardiac output DFI is expected to increase, and vice versa.^14^ According to current results of the regression analysis, DFI increased with increasing VTI, i.e. DFI increased with increasing cardiac function. This is contrary to what Simons et al. reported,^14^ and which is likely caused by the DFI being a patient specific parameter. Each patient has a unique cardiovascular anatomy requiring different cannula types and sizes,^18^ affecting pre- and afterload dependent centrifugal pump-based support flow and in turn the DFI value. Therefore, rather than comparing the DFI in relation to the VTI, it may be better to evaluate the intra-patient changes in DFI between full overall support and reduced overall support during a weaning trial.

Dynamic assessment methods in critical care patients have proven more valuable and useable in providing patient hemodynamic information.^19–21^ The changes in DFI (∆DFI, Figure 3) during the weaning trials employs such a dynamic approach. The results of the current study show that seven out of nine weaning trials support the expectation of the ∆DFI to not increase with diminished overall support when the VTI is improved (i.e. drainable volume does not increase, Figure 3, normal/improved load-responsiveness, lower right quadrant), and vice versa (Figure 3, blunted load-responsiveness, upper left quadrant). This shows the dynamic assessment of DFI itself, i.e. using ∆DFI to be a potential parameter for assessing on-pump drainable venous volume as a reflection of cardiac function. However, in order for the DFI to detect changes in drainable venous volume, the variability within a single measurement should be smaller than the difference observed within subsequent measurements. The average within-measurement IQR was 0.4 mL/rotation, which suggest changes in drainable volume smaller than 0.4 mL/rotation to be undetectable. Simons et al.^12^ also described that DFI cannot discriminate differences smaller than 0.5 mL/rotations within individuals. The current observed ∆DFI values are mostly smaller than the minimum detection range of 0.5 mL/rotations, implicating that these changes in DFI are difficult to discriminate. It can therefore be assumed that the DFI’s detection threshold is reached when assessing changes in drainable volume as seen in the weaning trials performed here.

The measurement of DFI requires manipulations of pump speed of sufficient magnitude and at well-defined time intervals.^12^ Possibly, pump speed alterations of ∼100 r/min superimposed on the overall support pump speed are not sufficient to supress variability within the measured flow values and detect changes in drainable volume during V-A ECLS. Simons et al. stated 100 r/min pump speed alterations to be sufficient to reliably measure DFI and suppress noise. These measurements were, however, performed in supported patients during cardiac surgery and with cardioplegia-induced cardiac arrest.^12^ In V-A ECLS, however, a beating heart results in hemodynamic pressure and volume fluctuations. Augmenting the amplitude of pump speed alterations would improve noise suppression during V-A ECLS, but would inevitably lead to undesirable larger alterations in overall support flow which would affect drainable volume itself. Therefore, additional investigations regarding the maximum safe but minimally required pump speed alteration are warranted. In addition, flow sensor accuracy could affect the DFI minimum detection range as well. With the accuracy of the flow sensor being ±0.3 L/min for flow ranges below 2.0 L/min and 15% for ranges above 2.0 L/min, a more accurate sensor might aid in decreasing the minimum DFI detection range.^22^

### Limitations

Some TEE recordings were of inferior quality, resulting in unobtainable VTI calculation and cardiac contractility assessment. Therefore, only visual interpretation of the cardiac function assessed by a cardiologist was feasible, resulting in a sample size of nine ∆VTI values from a total of 14 weaning trials.

## Conclusion

Even though the parameter’s current level of accuracy requires further investigation to increase reliability and possibly predictability, DFI seems likely to be a potential parameter in supporting TEE for the assessment of cardiac load-responsiveness.
